# GRK Mediates μ-Opioid Receptor Plasma Membrane Reorganization

**DOI:** 10.3389/fnmol.2019.00104

**Published:** 2019-05-01

**Authors:** Arisbel B. Gondin, Michelle L. Halls, Meritxell Canals, Stephen J. Briddon

**Affiliations:** ^1^Drug Discovery Biology Theme, Monash Institute of Pharmaceutical Sciences, Monash University, Melbourne, VIC, Australia; ^2^Division of Physiology, Pharmacology and Neuroscience, School of Life Sciences, Queen’s Medical Centre, University of Nottingham, Nottingham, United Kingdom; ^3^Centre of Membrane Proteins and Receptors, Universities of Birmingham and Nottingham, The Midlands, United Kingdom

**Keywords:** G protein-coupled receptor, μ-opioid receptor, G protein-coupled receptor kinase, plasma membrane, fluorescence correlation spectroscopy, fluorescence recovery after photobleaching

## Abstract

Differential regulation of the μ-opioid receptor (MOP) has been linked to the development of opioid tolerance and dependence which both limit the clinical use of opioid analgesics. At a cellular level, MOP regulation occurs via receptor phosphorylation, desensitization, plasma membrane redistribution, and internalization. Here, we used fluorescence correlation spectroscopy (FCS) and fluorescence recovery after photobleaching (FRAP) to detect and quantify ligand-dependent changes in the plasma membrane organization of MOP expressed in human embryonic kidney (HEK293) cells. The low internalizing agonist morphine and the antagonist naloxone did not alter constitutive MOP plasma membrane organization. In contrast, the internalizing agonist DAMGO changed MOP plasma membrane organization in a pertussis toxin-insensitive manner and by two mechanisms. Firstly, it slowed MOP diffusion in a manner that was independent of internalization but dependent on GRK2/3. Secondly, DAMGO reduced the surface receptor number and the proportion of mobile receptors, and increased receptor clustering in a manner that was dependent on clathrin-mediated endocytosis. Overall, these results suggest the existence of distinct sequential MOP reorganization events at the plasma membrane and provide insights into the specific protein interactions that control MOP plasma membrane organization.

## Summary Statement

G protein-coupled receptor kinase modulates μ-opioid receptor micro-diffusion at the plasma membrane prior to internalization.

## Introduction

The μ-opioid receptor (MOP) is the GPCR that mediates the analgesic effects of opioids such as morphine, fentanyl, and codeine. Despite being the mainstay analgesics for the treatment of acute pain, prolonged opioid use in inflammatory and chronic pain is severely limited by on-target adverse effects including tolerance and dependence. Furthermore, opioid prescription, abuse and overdose deaths have reached record levels globally (Rudd et al., 2016; Floyd and Warren, 2018), and the development of safer and more effective analgesics remains an unmet medical challenge. Differential regulation of MOP by morphine compared to other synthetic opioids or opioid peptides, has been linked to its increased propensity for tolerance and dependence (Duttaroy and Yoburn, 1995; Morgan and Christie, 2011; Williams et al., 2013); a better understanding of the molecular mechanisms governing MOP regulation is an important step in improving the therapeutic profiles of opioid analgesics.

As with other GPCRs, MOP-mediated signaling is initiated at the plasma membrane via protein–protein interactions between the receptor and its effectors. More recently, compartmentalization of GPCRs and their effectors within distinct subcellular locations or micro-domains of the plasma membrane has been shown to play a key role in their signaling (Sungkaworn et al., 2017; Eichel et al., 2018; Yanagawa et al., 2018). The concept that this may dictate specific cellular responses is particularly relevant in the context of MOP signaling, since MOP activation by different agonists results in distinct regulation and downstream signaling responses. For instance, the peptide agonist DAMGO causes multi-site phosphorylation of the receptor mediated by GRK2/3, inducing robust β-arrestin recruitment, and internalization. In contrast, the alkaloid agonist morphine causes limited receptor phosphorylation, and weak internalization (Doll et al., 2011; Lau et al., 2011; Just et al., 2013; Miess et al., 2018). MOP has been demonstrated to partition into lipid rafts and the dynamics of MOP diffusion at the plasma membrane contribute to the specific signaling responses elicited by different opioid ligands (Huang et al., 2007; Gaibelet et al., 2008; Zheng et al., 2008). For instance, the distribution of opioid receptors, including the MOP, into different nanoscale plasma membrane domains has been shown to be influenced by cholesterol (Rogacki et al., 2018), and MOP mobility, surface density, and the dynamics of plasma membrane lipids is affected by ethanol (Vukojevic et al., 2008b). Lateral mobility of MOP and MOP-G protein coupling is also changed differentially by the activating agonist (Sauliere-Nzeh Ndong et al., 2010) in a manner depending on the membrane cholesterol content (Melkes et al., 2016). Together, these studies suggest a link between distinct functional states of MOP and the dynamic organization of receptors within the plasma membrane. In addition, we have previously reported that DAMGO and morphine elicit different spatiotemporal signaling profiles, and that these are dictated by the lateral redistribution of MOP within the plasma membrane, rather than internalization (Halls et al., 2016). However, these previous studies lacked the temporal resolution required to investigate rapid diffusion changes of the receptor at the cell surface.

Live cell imaging techniques such as FCS and FRAP can provide greater temporal resolution to determine ligand-mediated changes in MOP dynamics and organization at the membrane. FCS is a highly sensitive confocal technique that can be used to quantify the diffusion and number of fluorescent species in small areas of living cells (Diekmann and Hoischen, 2014; Briddon et al., 2018). In FCS, fluorescent particles are excited as they pass through a small, defined confocal volume (∼0.2 fL) leading to time-dependent fluctuations in the detected fluorescence intensity; statistical analysis of these fluctuations using AC or PCH analysis allows the concentration and diffusion of fluorescent proteins to be determined in a small defined area (∼0.2 μm^2^) of the plasma membrane. FRAP can be used in conjunction with FCS to measure diffusion properties over a larger membrane area and give an indication of the proportion of mobile and immobile proteins (Haustein and Schwille, 2004; Pucadyil et al., 2007).

Here, we investigate the effect of different ligands on the organization and dynamics of MOP at the plasma membrane. Using FCS we quantify changes in the movement (diffusion coefficient; D_FCS_), number (particle number; N), and clustering (molecular brightness; 𝜀) of a fluorescently labeled SNAP-tagged MOP within small micro-domains of the membrane. Additionally, we use FRAP to determine the diffusion coefficient (D_FRAP_) and proportion of mobile (MF) receptors over a larger membrane area. We show that the internalizing agonist DAMGO slowed MOP diffusion in a time- and concentration-dependent manner, increased receptor clustering and the proportion of immobile MOP and reduced surface receptor number in a PTx -insensitive manner. These effects were not observed with the antagonist naloxone or the low internalizing agonist morphine. Notably, we were able to delineate the decrease in lateral mobility of MOP in response to DAMGO, which was dependent on GRK2/3 activity, from the clustering and decrease in MOP receptor number, which was dependent on clathrin-dependent endocytosis. These data provide insight into the role of GRKs as agonist-specific regulators of MOP micro-diffusion.

## Results

### Basal Plasma Membrane Organization of SNAP-MOP Detected by FCS and FRAP

To study the organization of MOP within living cell membranes, we performed FCS measurements on HEK293 cells stably expressing N-terminally SNAP-tagged human MOP (SNAP-MOP). Addition of the SNAP-tag at the N-terminus of the MOP did not alter its function, as shown by the ability of both DAMGO and morphine to inhibit forskolin-induced cAMP production in a similar way to the FLAG-tagged MOP (pEC_50_s: DAMGO = 7.67 ± 0.26 and 8.11 ± 0.08; morphine = 7.79 ± 0.31 and 7.92 ± 0.32 FLAG-MOP and SNAP-MOP, respectively, *n* = 4; Supplementary Figures 1A,B). Use of a SNAP-MOP fusion allowed specific labeling of cell surface MOP using a cell membrane impermeable SNAP-Surface^®^ 488 (BG-488) dye that specifically and covalently binds to SNAP-tagged proteins present at the cell surface.

**FIGURE 1:**
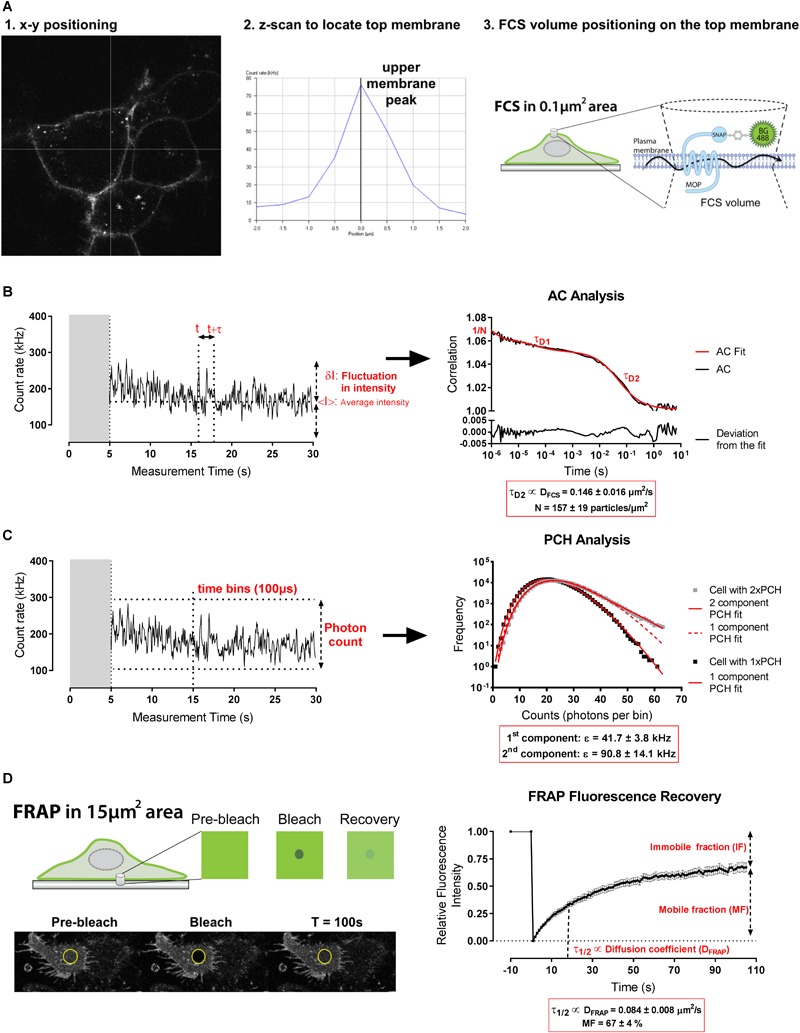
Basal plasma membrane organization of MOP detected by FCS and FRAP. **(A)** FCS measurement volume was positioned on the upper membrane using a live confocal image (1) and an intensity scan in z (2). (3) Schematic representation of FCS measurements on the membrane of HEK293 SNAP-MOP cells labeled with SNAP-Surface 488 (BG-488) dye. **(B)** Representative fluctuation trace in basal conditions for autocorrelation (AC) analysis in which fluctuations in intensity (δI) from the intensity mean (<I>) are calculated at two time points (t and t+τ) for all t and a range of *τ* values to generate an AC function that provides the average dwell time (τ_D_) and particle number (N). τ_D1_ represents the average dwell time of free BG-488 and was set to 32 μs; τ_D2_ represents the average dwell time of BG-488 bound to SNAP-MOP from which the receptor diffusion coefficient (D_FCS_; μm^2^/s) was calculated. N represents the number of particles and was used to calculate surface particle concentration (N/μm^2^). **(C)** Representative fluctuation trace in basal conditions and subsequent PCH analysis in which the amplitude of the fluctuations can be analyzed by quantifying the photons in defined time bins (100 μs). Super-Poissonian statistical analysis of the resulting frequency histogram allows the molecular brightness (𝜀) of SNAP-MOP containing particles to be determined. Two representative cell measurements under basal conditions are shown with the fit (solid red line) to one component (black squares) or two components (gray squares) accordingly. The poor 1 component fit (dotted red line) for the cell trace represented in gray squares is also shown. **(D)** Schematic representation of FRAP method and representative image of a cell in basal conditions during the bleaching protocol. Fluorescence recovery average trace of SNAP-MOP in basal conditions that provides a half-time (τ_1/2_) from which the diffusion coefficient (D_FRAP_; μm^2^/s), and mobile and immobile fractions (MF and IF, respectively) are inferred.

Fluorescence correlation spectroscopy measurements were performed on SNAP-MOP cells by positioning the confocal volume in *x*-*y* over the cell cytoplasm, and subsequently on the upper membrane at the peak intensity of a *z* scan (Figure 1A). FCS fluorescence fluctuation traces were recorded for 30s. The AC analysis yielded a two-component curve, consisting of a fast-diffusing component (τ_D1_; 10–15% of amplitude) indicative of residual free SNAP label with the remainder a slow component (τ_D2_) representing diffusion of the SNAP-MOP (see section “Materials and Methods”). The average dwell time (τ_D2_) and particle number (N) of the SNAP-MOP within the detection volume were obtained from the AC curve, from which the diffusion coefficient (D_FCS_; μm^2^/s), and receptor density (N/μm^2^) were calculated (Figure 1B and see section “Materials and Methods”). These measurements showed that under basal conditions, D_FCS_ for the SNAP-MOP was 0.146 ± 0.016 μm^2^/s with a receptor density (N) of 157 ± 19 particles/μm^2^ (*n* = 14 cells) (Figure 1B). Analysis of the same fluorescence fluctuations using PCH analysis yielded the average molecular brightness (𝜀; counts per molecule per second, kHz) of the fluorescent species (Figure 1C and Materials and Methods), providing an indication of the extent of SNAP-MOP clustering. Under basal conditions PCH analysis of fluctuations from the majority (81%) of cells fitted to a single brightness component with an average 𝜀 of 41.7 ± 3.8 kHz (*n* = 21 cells) (Figure 1C). Interestingly, in 19% of the cells analyzed, a second brighter component (average 𝜀 = 90.8 ± 14.1 kHz) was detected (Figure 1C), indicating the presence of higher-order oligomeric forms of SNAP-MOP in basal conditions. Of note, the brighter component was always less abundant relative to the single component.

In order to ensure that accurate diffusion coefficients were obtained, excitation laser power was optimized to give a maximum signal to noise ratio (highest 𝜀) with minimal spot photobleaching (indicated by no increase in D_FCS_ or decrease in N) (Supplementary Figure 1C). On the basis of these data, subsequent experiments used a laser power of ∼0.08 kW/cm^2^. FCS measurements were also conducted on cells labeled with a range of BG-488 concentrations that ensured that all of the cell surface receptors had been labeled (saturated N) whilst minimizing the amount of free BG-488 label left after washing. At concentrations of BG-488 above 50 nM, the particle number (N) (Supplementary Figure 1D) and molecular brightness (𝜀) remained constant (Supplementary Figure 1E), indicating labeling of all cell surface receptors. On the basis of these data, subsequent experiments used 200 nM BG-488 label. It was also noted that as the concentration of BG-488 label increased, the number of cells that required a second component for the PCH fit also increased (Supplementary Figure 1E).

Since FCS only measures mobile receptor population, FRAP was performed over a larger area of the lower cell membrane of adherent cells to determine a macro diffusion coefficient (D_FRAP_), as well as the proportions of mobile (MF) and immobile (IF) receptors (Figure 1D). Using a circular bleach area with radius of 2.2 μm (area ∼ 15 μm^2^), FRAP measurements showed that under basal conditions 67 ± 4% of the receptor population is mobile over this area with a diffusion rate (D_FRAP_) of 0.084 ± 0.008 μm^2^/s which was slower than that measured for FCS (D_FCS_).

### Ligand-Induced Changes in the Plasma Membrane Organization of SNAP-MOP

**FIGURE 2:**
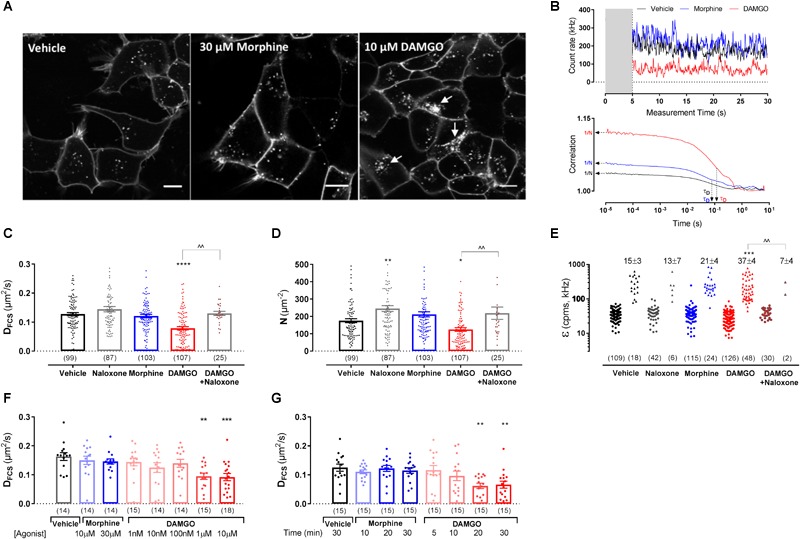
Fluorescence correlation spectroscopy measurements in HEK293 SNAP-MOP cells following ligand stimulation. **(A)** Representative images from HEK293 SNAP-MOP cells labeled with SNAP-Surface 488 (BG-488) dye after stimulation with vehicle, 30 μM morphine or 10 μM DAMGO for 20 min. Scale bar 10 μm; arrows represent agonist-induced internalization. **(B)** Representative FCS traces from single HEK293 SNAP-MOP cells after stimulation with vehicle, 30 μM morphine, or 10 μM DAMGO for 20 min (gray area indicates the initial 5 s of data that were removed, see section “Materials and Methods”), and their corresponding AC fit indicating N and τ_D_ of SNAP-MOP. **(C)** Diffusion coefficient of SNAP-MOP upon stimulation with 30 μM naloxone, 30 μM morphine, 10 μM DAMGO (20 min, 37°C), or 10 μM DAMGO after pre-treatment with 30 μM naloxone (30 min, 37°C) (*n* = 25–107 cells from 5 to 23 independent experiments; one-way ANOVA, *F*(df1,df2): *F*(4,416) = 15.04, *P* < 0.0001). **(D)** Particle number of SNAP-MOP upon stimulation with 30 μM naloxone, 30 μM morphine, 10 μM DAMGO (20 min, 37°C), or 10 μM DAMGO after pre-treatment with 30 μM naloxone (30 min, 37°C) (*n* = 25–107 cells from a minimum of 5 independent experiments; *F*(4,416) = 11.06, *P* < 0.0001). **(E)** Molecular brightness of SNAP-MOP after stimulation with 30 μM naloxone, 30 μM morphine, 10 μM DAMGO, or 10 μM DAMGO after pre-treatment with 30 μM naloxone. For each condition, brightness values for the first (left) and second (right) component are shown [*n* = 30–126 cells from a minimum of 6 independent experiments; *F*(4,82) = 5.74, *P* < 0.0001], with the percentage of cells requiring a two-component fit annotated. **(F)** Diffusion coefficient of SNAP-MOP upon increasing concentration of morphine or DAMGO after 20 min incubation [*n* = 14–18 cells from 3 independent experiments; *F*(7,110) = 4.14, *P* < 0.0001]. **(G)** Diffusion coefficient of SNAP-MOP upon stimulation with 30 μM morphine or 10 μM DAMGO with increasing time incubation periods [*n* = 15 from 3 independent experiments; *F*(7,113) = 4.36, *P* = 0.0003]. Each data point on the scatter plots represents a measurement from an individual cell (number of cells in parenthesis) and column plots represent mean ± SEM of the indicated number of cells. ^∗^ denotes significance vs. vehicle treatment and ^∧^ denotes significance vs. DAMGO control in one-way ANOVA with Sidak’s multiple comparisons test (^∗^*P* < 0.05, ^∗∗^*P* < 0.01, ^∗∗∗^*P* < 0.001, ^∗∗∗∗^*P* < 0.0001, ^∧∧^*P* < 0.01).

The effect of ligand stimulation on SNAP-MOP membrane organization was then assessed using FCS and FRAP. Substantial fluorescence remained at the plasma membrane after stimulation with saturating concentrations of the poorly internalizing agonist morphine (30 μM) or the internalizing agonist DAMGO (10 μM) for 20 min (Figure 2A). This is not surprising since we have previously shown that DAMGO-induced internalization reached a maximum 1 h after agonist stimulation (Halls et al., 2016; Miess et al., 2018). We then recorded FCS fluctuation traces of SNAP-MOP following exposure to each ligand and performed AC and PCH analysis (Figure 2B). Stimulation with DAMGO (10 μM) caused a significant decrease in MOP diffusion co-efficient (D_FCS_) (*P* < 0.0001, ANOVA and *post hoc* Sidak’s test; n numbers and ANOVA parameters given in figure legends for this and all subsequent *P* values) (Figure 2C and Table 1), a decrease in particle number (N) (*P* = 0.032) (Figure 2D and Table 1), and DAMGO stimulation increased the percentage of cells with a bright second component in PCH analysis (from 15% in vehicle to 37% in DAMGO-treated cells; *P* = 0.0008), suggesting that DAMGO stimulation induces clustering of SNAP-MOP (Figure 2E and Table 1). These changes were mediated by receptor activation, since they were not present in cells which were pre-treated with the MOP antagonist naloxone (10 μM) (*P* = 0.001, 0.008, and 0.004 for differences in D_FCS_, N and clustering in presence and absence of naloxone, respectively). The slowing in diffusion caused by DAMGO was concentration- (Figure 2F) and time-dependent (Figure 2G), with a significant decrease in D_FCS_ seen at concentrations of 1 μM and above (*P* = 0.003), which is consistent with DAMGO’s potency for recruitment of regulatory proteins (Miess et al., 2018), and after 20 min of stimulation, which is prior to internalization (Miess et al., 2018).

**Table 1:** Diffusion coefficient (D_FCS_), particle concentration (N) and clustering measured by FCS and diffusion coefficient (D_FRAP_) and immobile fraction (IF) measured by FRAP of SNAP-MOP expressing cells under different treatment conditions.

Treatment conditions		FCS			FRAP	
		D_FCS_ (μm^2^/s)	*N* (particles/μm^2^)	Clustering (% cells)	D_FRAP_ (μm^2^/s)	IF (%)
	Vehicle	0.128 ± 0.005	175 ± 11	15 ± 3	0.084 ± 0.008	34 ± 3
	Naloxone	0.145 ± 0.009	245 ± 17**	13 ± 7	0.089 ± 0.007	33 ± 2
**Control**	Morphine	0.121 ± 0.006	212 ± 15	21 ± 4	0.079 ± 0.005	33 ± 3
	DAMGO	0.079 ± 0.006§	125 ± 10*	37 ± 4***	0.080 ± 0.007	52 ± 4§
	DAMGO + Naloxone	0.130 ± 0.007^∧∧^	219 ± 35^∧∧^	7 ± 4^∧∧^	ND	ND

	Vehicle	0.132 ± 0.012	155 ± 23	20 ± 0	ND	ND
**PTx**	Naloxone	0.127 ± 0.013	241 ± 25	0 ± 0	ND	ND
	Morphine	0.122 ± 0.006	236 ± 20	0 ± 0	ND	ND
	DAMGO	0.093 ± 0.010#	170 ± 18	30 ± 13	ND	ND

	Vehicle	0.129 ± 0.012	112 ± 9	15 ± 6	0.105 ± 0.014	45 ± 4
**Pitstop2**	Morphine	0.126 ± 0.011	204 ± 19	21 ± 10	0.132 ± 0.027	24 ± 3#
	DAMGO	0.082 ± 0.010#	143 ± 16	21 ± 9	0.113 ± 0.031	48 ± 4

	Vehicle	0.126 ± 0.011	180 ± 19	13 ± 8	0.102 ± 0.013	40 ± 5
**Cmpd101**	Morphine	0.118 ± 0.010	217 ± 23	11 ± 4	0.085 ± 0.009	28 ± 3
	DAMGO	0.106 ± 0.008	243 ± 26	13 ± 5	0.084 ± 0.012	30 ± 4

*^∗^denotes significance vs. vehicle control (^∗^*P* < 0.05, ^∗∗^*P* < 0.01, ^∗∗∗^*P* < 0.001, ^x^*P* < 0.0001) in one-way ANOVA with Sidak’s multiple comparisons test. ^∧∧^denotes significance vs. DAMGO control (^∧∧^*P* < 0.01) in one-way ANOVA with Sidak’s multiple comparisons test. ^#^denotes significance vs. vehicle in the same inhibitor pre-treatment condition (^#^*P* < 0.05) in one-way ANOVA with Sidak’s multiple comparisons test. ND, not determined. All data are shown as mean ± SEM. For clarity, n numbers (cells and independent experiments) are referred to in the appropriate figures and their legends but are from a minimum of 5 independent experiments.*

In contrast to DAMGO, stimulation with morphine (up to 30 μM) or naloxone alone (30 μM) did not cause any significant changes in D_FCS_ or clustering compared to vehicle treatment (P = 0.91 and 0.24, respectively; Figure 2C–G), indicating that changes in MOP plasma membrane organization are agonist-specific. Interestingly, incubation with naloxone alone significantly increased the particle number (N) compared to vehicle treatment (*P* = 0.002) (Figure 2D). This increase might indicate that naloxone prevents constitutive MOP internalization observed in vehicle (Figure 2A), resulting in an increase in surface receptor number.

**FIGURE 3:**
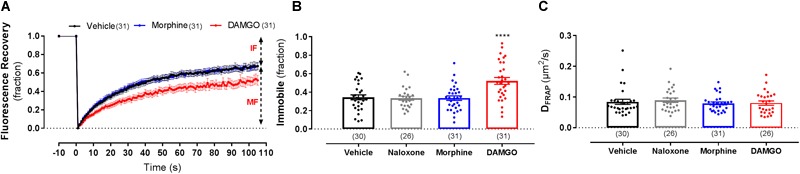
Fluorescence recovery after photobleaching measurements on the lower membrane of HEK293 SNAP-MOP cells following ligand stimulation. **(A)** Fluorescence recovery curves of HEK293 SNAP-MOP cells during FRAP experiments under control conditions and following stimulation with 30 μM morphine or 10 μM DAMGO for 20 min at 37°C (*n* = 26–31 from 6 independent experiments). MF, mobile fraction and IF, immobile fraction. **(B)** Immobile fraction and **(C)** diffusion coefficient of SNAP-MOP as determined by FRAP (D_FRAP_) analysis following stimulation with 30 μM naloxone, 30 μM morphine or 10 μM DAMGO (*n* = 26–31 from 6 independent experiments; one-way ANOVA, *F*(df1,df2): *F*(3,114) = 10.48, *P* < 0.0001). Each data point of the scatter plots represents an individual cell measurement (number of cells in parenthesis) and column plots represent mean ± SEM of the indicated number of cells. ^∗^ denotes significance vs. vehicle treatment in one-way ANOVA with Sidak’s multiple comparisons test (^∗∗∗∗^*p* < 0.0001).

Since FCS can only determine the properties of mobile receptors, we also used FRAP to assess whether ligand stimulation changes the proportion of mobile vs. immobile receptors (Figure 3A). Following exposure of cells to 10 μM DAMGO, the immobile fraction (IF) of MOP was significantly increased from 34 ± 3% in vehicle-treated cells to 52 ± 4% in DAMGO-treated conditions (*P* < 0.0001) (Figure 3B). In contrast, treatment with morphine or naloxone caused no change in mobile fraction compared to vehicle (*P* = 0.99 and 0.99, respectively). Contrary to the decrease in D_FCS_ induced by DAMGO, D_FRAP_ remained unchanged upon stimulation with any of the ligands (Figure 3C).

These data therefore suggest ligand-specific changes in MOP membrane organization, with DAMGO stimulation slowing MOP diffusion, reducing surface receptor number, increasing receptor clustering, and the proportion of immobile receptors. While these effects were reversed with naloxone, none of these changes were observed following stimulation with morphine.

### MOP Membrane Reorganization Is Independent of G_i/o_ Protein Activation

**FIGURE 4:**
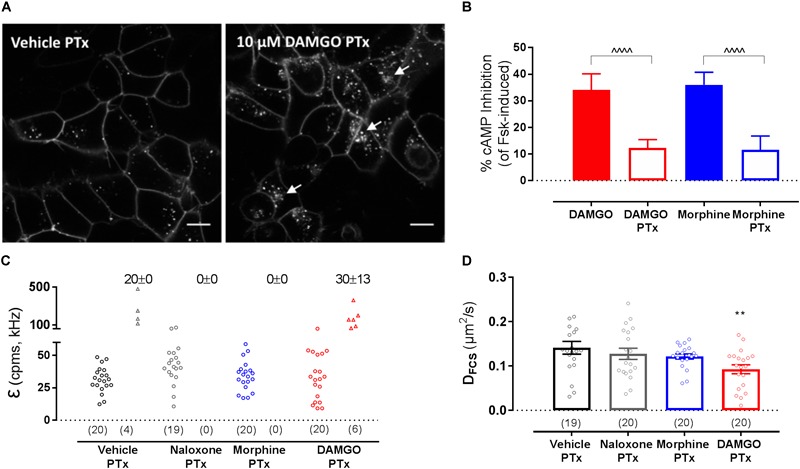
Effect of pertussis toxin (PTx) pre-treatment on SNAP-MOP function and membrane organization. HEK293 SNAP-MOP cells were exposed to PTx (100 ng/ml overnight, 37°C). **(A)** Representative images of PTx pre-treated HEK293 SNAP-MOP cells after stimulation with vehicle or 10 μM DAMGO. Scale bar 10 μm, arrows represent agonist-induced internalization. **(B)** Agonist-mediated inhibition of forskolin-induced cAMP accumulation upon stimulation with 10 μM DAMGO or 10 μM morphine in control (closed bars) or PTx pre-treated cells (open bars) in a CAMYEL BRET assay. Data are shown as percentage of inhibition of cAMP induced by 10 μM forskolin and represent mean ± SEM of 3 independent experiments (one-way ANOVA, *F*(df1,df2): *F*(3,12) = 30.17, *P* < 0.0001). ^∧^ denotes significance vs. DAMGO or morphine in the untreated condition (^∧∧∧∧^*P* < 0.0001) with Sidak’s multiple comparisons test. **(C)** Molecular brightness [*F*(3,12) = 5.4, *P* = 0.014] and **(D)** diffusion coefficients from FCS measurements of SNAP-MOP after stimulation with 30 μM naloxone, 30 μM morphine or 10 μM DAMGO in PTx pre-treated cells (*n* = 19–20 cells from 4 independent experiments, *F*(7,132) = 4.59, *P* = 0.0001). For each condition, brightness values for the first (left) and second (right) component are shown, with the number of cells requiring a two-component fit indicated as percentage. Each data point on the scatter plots represents a measurement from an individual cell (number of cells in parenthesis) and column plots represent mean ± SEM of the indicated number of cells. ^∗^ denotes significance vs. vehicle treatment in one-way ANOVA with Sidak’s multiple comparisons test (^∗∗^*P* < 0.01).

The dependence of MOP reorganization on G protein activation was then investigated using PTx as a Gα_i/o_ inhibitor (100 ng/ml, overnight treatment). Consistent with previous data (Halls et al., 2016), inhibition of Gα_i/o_ activation did not prevent DAMGO-induced MOP internalization (Figure 4A) at a PTx concentration that was effective at preventing agonist-induced adenylyl cyclase inhibition (Figure 4B). However, PTx treatment did not affect DAMGO-induced clustering (Figure 4C and Table 1) or slowing in diffusion (Figure 4D and Table 1). Altogether, these data suggest that G protein activation is not necessary for the changes in MOP membrane organization induced by DAMGO.

### Effect of Internalization Inhibition on Ligand-Induced Changes in MOP Organization

**FIGURE 5:**
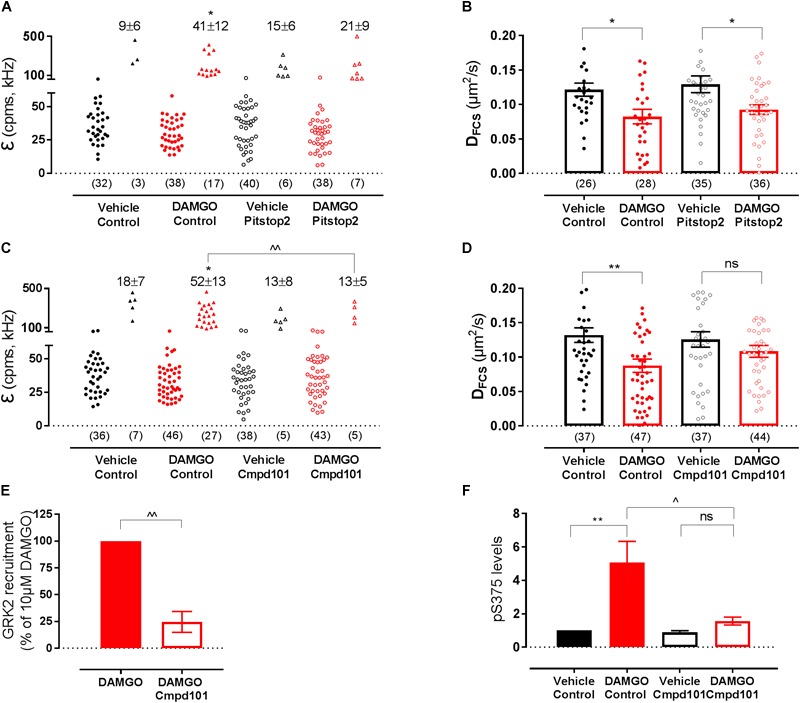
Effect of internalization inhibitor Pitstop2 and GRK2/3 inhibitor Cmpd101 on SNAP-MOP membrane organization. HEK293 SNAP-MOP cells were exposed to the internalization inhibitor Pitstop2 (30 μM, 30 min, 37°C) or GRK2/3 inhibitor Cmpd101 (30 μM, 30 min, 37°C). **(A)** Molecular brightness of SNAP-MOP after stimulation with vehicle or 10 μM DAMGO in control or Pitstop2 pre-treated cells [*n* = 32–40 cells from 6 to 8 independent experiments, one-way ANOVA, *F*(df1,df2): *F*(3,26) 2.67, *P* = 0.060]. **(B)** Diffusion coefficient of SNAP-MOP upon stimulation with vehicle or 10 μM DAMGO in control or Pitstop2 pre-treated cells [*n* = 26–36 from 6 to 8 independent experiments, *F*(3,121) = 5.00, *P* = 0.003]. **(C)** Molecular brightness of SNAP-MOP after stimulation with vehicle or 10 μM DAMGO in control or Cmpd101 pre-treated cells [*n* = 36–46 cells from 6 to 8 independent experiments, *F*(3,28) = 4.86, *P* = 0.008]. **(D)** Diffusion coefficient of SNAP-MOP upon stimulation with vehicle or 10 μM DAMGO in control or Cmpd101 pre-treated cells [*n* = 37–47 cells from 6 to 8 independent experiments, *F*(3,161) = 4.15, *P* = 0.007]. For each condition of the molecular brightness data, brightness values for the first (left) and second (right) component are shown, with the number of cells requiring a two-component fit indicated as percentage. Each data point on the scatter plots represents a measurement from an individual cell (number of cells in parenthesis) and column plots represent mean ± SEM of the indicated number of cells or individual experiments. **(E)** HEK293 were transiently transfected with FLAG-MOP-NLuc and GRK2-Venus to measure GRK2 recruitment in a BRET assay after stimulation with 10 μM DAMGO in control or Cmpd101 pre-treated cells (*n* = 3 independent experiments). The BRET ratio of vehicle-treated cells was subtracted, data represent mean ± SEM normalized to control condition, ^∧∧^ denotes significance vs. DAMGO alone (*P* = 0.002, unpaired Student’s *t*-test). **(F)** Quantification of pS375 FLAG-MOP upon stimulation with vehicle or 1 μM DAMGO in control or Cmpd101 pre-treated cells. Phosphorylation of S375 was quantified as the ratio of anti-phosphoS375 (pS375) MOP site antibody immunostaining divided by FLAG immunostaining and normalized to vehicle of the control condition [*n* = 3 independent experiments, *F*(3,8) = 9.348, *P* = 0.005]. ^∗^ denotes significance vs. vehicle treatment and ^∧^ denotes significance vs. DAMGO control in one-way ANOVA with Sidak’s multiple comparisons test (ns, not significant, *P* > 0.05, ^∗^*P* < 0.05, ^∗∗^*P* < 0.01, ^∧^*P* < 0.05, ^∧∧^*P* < 0.01).

To investigate whether the DAMGO-mediated changes in MOP organization were dependent on MOP internalization, we used an inhibitor of clathrin-dependent internalization, Pitstop2 (30 μM, 30 min pre-treatment), which has previously been shown to block DAMGO-induced MOP endocytosis (Halls et al., 2016). In FCS experiments, Pitstop2 prevented DAMGO-induced receptor clustering as shown by the reduction in the percentage of cells that showed the second brighter PCH component in the presence of the inhibitor (*P* = 0.944 DAMGO/Pitstop2 vs. Pitstop2 alone) (Figure 5A). There was also no DAMGO-mediated increase in immobile fraction detected using FRAP in the presence of Pitstop2 (*P* = 0.95) (Table 1). However, inhibiting internalization did not significantly affect the DAMGO-induced slowing in diffusion measured by FCS (*P* = 0.028 DAMGO/Pitstop2 vs. Pitstop2 alone) (Figure 5B and Table 1). Altogether, these data suggest that MOP diffuses laterally at the plasma membrane, clustering and immobilizing in clathrin-coated pits prior to internalization. Some MOP organization events such as clustering are dependent on clathrin-mediated endocytosis. However, Pitstop2 was not able to prevent the DAMGO-induced decrease in D_FCS_, highlighting that a different molecular mechanism must be underlying this micro-diffusion event.

### Effect of GRK Inhibition on Ligand-Induced Changes in MOP Organization

We then investigated a potential role for GRKs in MOP reorganization since interaction with and phosphorylation by these kinases occurs rapidly after receptor activation, prior to internalization and in an agonist-dependent manner (Miess et al., 2018). As expected from an event that precedes internalization, inhibition of GRK2/3 with Cmpd101 (30 μM, 30 min pre-treatment) prevented DAMGO-induced clustering measured with FCS (*P* = 0.003 DAMGO vs. DAMGO/Cmpd101; *P* > 0.999 DAMGO/Cmpd101 vs. Cmpd101 alone; Figure 5C and Table 1) and the increase in immobile fraction detected by FRAP (*P* = 0.442; Table 1). Remarkably, and unlike PitStop2, Cmpd101 also prevented the DAMGO-induced slowing as measured by decrease in D_FCS_ (*P* = 0.550 DAMGO/Cmpd101 vs. Cmpd101 alone; Figure 5D and Table 1). Cmpd101 treatment significantly reduced GRK2-Venus recruitment to FLAG-MOP-NLuc (Figure 5E) and phosphorylation at one of the key C-terminal phosphorylation residues S375 (Figure 5F), indicating that Cmpd101 prevents both recruitment and subsequent activity of GRK2 at the MOP. These data suggest the existence of a GRK-dependent mechanism (scaffolding or phosphorylation), which results in the DAMGO-stimulated slowing in diffusion and precedes receptor clustering and internalization.

## Discussion

The formation of highly dynamic signaling complexes at the plasma membrane of the cell is key for the generation of specific cellular responses to extracellular stimuli. Stimulation of transmembrane proteins such as GPCRs can alter the composition of these receptor-effector platforms to generate a tailored signaling profile. This is illustrated, for example, by the process of receptor internalization, where prolonged stimulation of a GPCR by an agonist leads to phosphorylation of its intracellular domains which results in the recruitment of a myriad of regulatory proteins (including β-arrestins, AP-2, clathrin). These proteins facilitate receptor accumulation in clathrin-coated pits and trafficking of the receptor to intracellular compartments. While receptor endocytosis represents a macroscopic change in receptor reorganization, diffusion of adaptor proteins at the microscopic scale has been suggested to occur prior to internalization (Rappoport et al., 2006). Recent advances in quantitative live cell imaging techniques and receptor labeling have been instrumental in providing further information on these dynamic micro-changes in receptor organization that occur at the plasma membrane prior to receptor accumulation into intracellular compartments (Sungkaworn et al., 2017; Yanagawa et al., 2018). In addition, such studies have also demonstrated that effector proteins such as adenylyl cyclase redistribute and reorganize their micro-environment to generate highly specialized signaling hubs (Ayling et al., 2012).

Understanding such microdomain level diffusion events is of particular relevance for the MOP, since activation of MOP by different ligands results in distinct regulatory profiles. While endogenous opioid peptides and their analogs induce robust receptor internalization, other opioid ligands such as morphine are very weak at driving MOP internalization (Keith et al., 1996; Whistler and von Zastrow, 1998). Moreover, we have recently shown that differential activation of MOP also results in distinct spatiotemporal signaling profiles that are controlled by a change in distribution of the receptor within the plasma membrane (Halls et al., 2016). Here, we have used complementary imaging techniques (FCS and FRAP) to gain further understanding of the changes in plasma membrane MOP distribution that occur following receptor activation with DAMGO (an enkephalin derivative that causes robust MOP internalization) compared to morphine (a poor internalizing agonist).

Our data shows that only DAMGO, but not morphine, can change the lateral organization of the MOP from basal conditions in a concentration and time-dependent manner, prior to its movement to clathrin-coated pits and internalization. We detected significant DAMGO-induced slowing in diffusion at 1 μM after 20 min of stimulation, a time point at which internalization is minimal (Halls et al., 2016). For this reason, we anticipate that this slowing in diffusion alludes to an event prior to receptor endocytosis. DAMGO-induced reorganization occurs at two different levels; there is a micro-reorganization event (reflected in changes in the diffusion coefficient) and macro-reorganization event (which changes surface receptor number, clustering and immobile fraction). Differences between micro- and macro-level MOP organization were also illustrated by the slower MOP diffusion co-efficient determined by FRAP compared to FCS. This can be interpreted as a free local diffusion within a confined domain, but restricted movement across larger distances. Notably, DAMGO-induced changes were only seen in D_FCS_, but not in D_FRAP_, consisting with a local change in organization.

**FIGURE 6:**
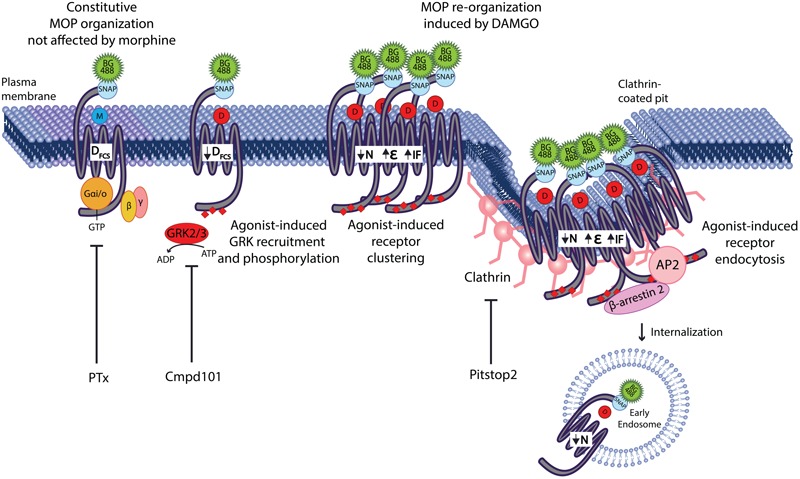
GRK2 mediates MOP micro-diffusion events that precede internalization. Agonist stimulation of SNAP-MOP with morphine does not alter its constitutive plasma membrane organization. In contrast, agonist stimulation with DAMGO causes re-organization of SNAP-MOP indicated by the decrease in diffusion coefficient (D_FCS_), the decrease in particle concentration (N), the increase in molecular brightness (𝜀), and the increase in immobile fraction (IF). G protein activation is not involved in SNAP-MOP plasma membrane organization, whereas GRK2/3 activity mediates DAMGO-induced slowing in D_FCS_ independent or prior to clustering and movement of SNAP-MOP into clathrin-coated pits to proceed toward internalization. The latter events are responsible for changes in macro-scale parameters such as the reduced N, increased 𝜀 and IF.

Additionally, we showed that the plasma membrane reorganization induced by DAMGO is independent of G protein activation and that the “macro” changes in MOP clustering are mediated by clathrin-dependent endocytosis. Interestingly, the initial slowing of the MOP was only prevented by Cmpd101, a GRK2/3 inhibitor, but was independent of (or occurs prior to) clathrin-mediated endocytosis. This suggests that the large-scale changes induced by internalization such as the decrease in particle number and the increase in molecular brightness represent clustering into coated pits where the receptor is immobilized within the clathrin lattice. We propose that a rapid reorganization event that involves a decrease of the diffusion of MOP occurs at the plasma membrane prior to internalization. Such lateral reorganization requires GRK2 and is likely to be responsible for the distinct agonist-specific signaling patterns that we have previously observed (as per Halls et al., 2016). This reorganization is followed by larger scale movements of the MOP into clathrin-coated pits resulting in clustering and endocytosis (Figure 6).

DAMGO-induced slowing in MOP diffusion could be caused by a change in the molecular composition of the receptor complex (i.e., direct interaction with GRKs, although interaction with other phosphorylation-dependent proteins cannot be dismissed) soon after receptor activation and prior to its accumulation in clathrin-coated pits. A change in the composition of the MOP complex on this timescale would be consistent with our previous observations of a DAMGO-induced receptor redistribution to control transient activation of cytosolic and nuclear ERK (Halls et al., 2016). Therefore, not only is the differential recruitment of GRK2/3 by DAMGO vs. morphine important for MOP regulation (Miess et al., 2018), it also plays an essential role in MOP diffusion events that facilitate activation of compartmentalized signaling, revealing a novel role of GRKs as agonist-specific regulators of MOP plasma membrane signaling. On that note, GRKs are typically recognized for their catalytic activity in mediating agonist-induced phosphorylation of GPCRs that eventually result in receptor internalization. Importantly, scaffolding roles for GRKs have also been described in which the kinase participates in the generation of a macromolecular signalosomes in a manner that can be independent of its kinase activity (Penela et al., 2010). The mechanism underlying this GRK-dependent micro-diffusion event of MOP remains to be elucidated and future studies should investigate whether it is the kinase or the scaffolding function of GRK2 that is required for the observed changes in plasma membrane reorganization of MOP.

Several studies have shown the complexity of MOP dynamics within the plasma membrane. Recent FCS and PALM studies have provided unprecedented details on the dynamic lateral organization of MOP and KOP. These studies have shown that in unstimulated cells, GFP tagged receptors are organized into nano-domains that partially overlap with cholesterol-rich domains and are excluded from GM1-ganglioside-enriched domains (Rogacki et al., 2018). However, this study did not report on the changes that may occur upon receptor stimulation. Previous FRAP studies using a GFP-MOP have investigated the lateral diffusion of MOP upon DAMGO and morphine stimulation (Sauliere-Nzeh Ndong et al., 2010). While morphine seemed to induce limited diffusion, with small domain size and diffusion coefficient, DAMGO displayed bigger changes in diffusion range in addition to the effects observed with morphine. Interestingly, these latter long-range changes were absent when receptor endocytosis was inhibited, and the small-range changes seemed to be dependent on G protein activation. Although we did not observe changes in FRAP diffusion coefficients, our FRAP data, showing that upon treatment with Pitstop2 the immobile receptor fraction of DAMGO-activated MOP is not different than vehicle, is in line with these observations. A second FRAP study has shown ligand-dependent changes in the diffusion rate of MOP that are differentially affected by cholesterol depletion (Melkes et al., 2016). Such divergent results concerning the influence of agonists on the diffusion coefficients of MOP can, be attributed to different experimental conditions including the use of different fusion proteins. The attachment of a fluorescent protein tag to the receptor does not allow to distinguish between receptors that are already at the cell surface at the time of stimulation and newly synthesized receptors that are subsequently incorporated into the plasma membrane from the intracellular compartment, thus properties from different receptor pools might have been incorporated. Moreover, addition of the fluorescent protein within the C-terminus could interfere with complex formation and could affect the diffusion rates measured.

Experimental and computational evidence has also highlighted that the membrane organization of MOP can be influenced by changes in lipid content (Vukojevic et al., 2008a; Marino et al., 2016). Cholesterol has been shown to promote MOP homodimerization (Zheng et al., 2012), agonist binding (Gaibelet et al., 2008), coupling with G proteins (Gaibelet et al., 2008; Zheng et al., 2012), and translocation of β-arrestin (Qiu et al., 2011). Such movement between membrane domains with different lipid composition (e.g., cholesterol/non-cholesterol) could account for the slowing in MOP diffusion induced by DAMGO although further experiments should address the role of GRKs in this event. Nevertheless, this provides a potential mechanism by which the direct interaction of lipids with the receptor or the membrane micro-environment can facilitate interactions with specific signaling effectors and account for the agonist-specific spatiotemporal signaling elicited by the MOP (as per Halls et al., 2016).

In summary, we have described here that the plasma membrane reorganization of MOP at the micro scale level is dependent on GRK2/3 and at the macro scale level is dependent on clathrin-dependent internalization. This study therefore reveals an important and novel role of GRKs in modulating plasma membrane MOP organization; and provides evidence that the lateral diffusion of MOP represents a molecular event, distinct and prior to internalization, that is differentially regulated by opioids and controls its spatiotemporal signaling.

## Materials and Methods

### Reagents

D-Ala^2^,N-Me-Phe^4^,Gly^5^-ol-enkephalin was obtained from Mimotopes. Morphine, Rhodamine 6G and M2-anti-FLAG were from Sigma-Aldrich (Gillingham, Dorset, United Kingdom). Naloxone was from Tocris. SNAP-Surface 488 was from New England Biolabs (Ipswich, MA, United States). The antibody anti-pS375 was from Cell Signaling. Secondary antibodies (raised in donkey) conjugated to Alexa-Fluor 488 or 647 were from Jackson ImmunoResearch. Coelenterazine h was from NanoLight. Furimazine was from Promega. Compound 101 was from HelloBio. Pitstop2 was from Abcam. PTx was from Millipore.

### Plasmids

To create the SNAP-MOP constructs, the full coding sequence for the human MOP (hMOP) was ligated into a pcDNA3.1(+) vector containing the 5-HT_3_ receptor membrane localization signal sequence and the SNAP-tag (New England Biolabs, Ipswich, MA, United States) (Gherbi et al., 2015) and a neomycin resistance gene. Initial site-directed mutagenesis was required to remove the internal BamHI site in the MOP receptor while maintaining the amino acid sequence. Additional site-directed mutagenesis was performed to mutate the start codon (Met to Leu) on the MOP cDNAs. These were then ligated to the C-terminus of SNAP using BamHI and XhoI restriction enzymes. The resulting fusion protein contained a Gly-Ser linker between the SNAP open reading frame (ORF) and the MOP ORF.

To create the FLAG-MOP-NLuc, the NLuc sequence (Soave et al., 2016) was ligated immediately after the C-terminus of FLAG-mMOP cDNA without stop codon (Miess et al., 2018) with XhoI and XbaI restriction enzymes in a pcDNA3.1 vector. All sequences were confirmed by DNA sequencing.

GRK2-Venus was from D. Jensen (Columbia University, New York), CAMYEL sensor has been previously characterized (Jiang et al., 2007).

### Generation of Cell Lines

Human embryonic kidney cells (ATCC, Middlesex, United Kingdom) were grown in Dulbecco’s modified Eagle’s medium (DMEM) supplemented with 10% (v/v) fetal calf serum and maintained at 37°C in a humidified incubator containing 5% CO_2_. Cells were transfected with the pcDNA3.1+ vector containing SNAP-MOP constructs using Fugene HD (Promega) as transfection reagent according to the manufacturer’s instructions. Twenty-four hours post transfection, the medium was supplemented with 1 mg/ml G418-selective pressure for 2 weeks for the generation of stable mixed population HEK293 SNAP-hMOP cell line.

### Cell Plating and Treatments

Stable mixed population cell lines were plated onto poly-D-lysine–coated 8-well Labtek No.1 borosilicate chambered coverglasses (Nunc Nalgene International, Thermo Fisher Scientific). On the day of the experiment, SNAP tag labeling was performed by incubating cells with 200 nM (or as otherwise indicated) SNAP-Surface 488 (BG-488) dye in fresh cell culture media for 30 min at 37°C. Cells were washed in pre-warmed HEPES-buffered saline solution (HBSS; Briddon et al., 2004) containing 4.5 mM D-glucose and pretreated with inhibitors for 30 min at 37°C, except for pertussis toxin (PTx; 16 h pre-treatment). Inhibitors were used at the following concentrations: 30 μM Pitstop2, 30 μM Cmpd101, or 100 ng/ml PTx. Cells were then stimulated for 20 min (or as otherwise indicated) at 37°C with vehicle (0.3% v/v DMSO), 10 μM DAMGO, 30 μM morphine, 30 μM naloxone, or 10 μM DAMGO after pre-treatment for 30 min at 37°C with 30 μM naloxone.

### Fluorescence Correlation Spectroscopy (FCS)

Cells were equilibrated to room temperature (22 ± 2°C) to minimize artifacts from temperature-induced plasma membrane fluctuations. FCS measurements were taken at room temperature on a Zeiss LSM510NLO Confocor 3 inverted confocal microscope using a 40× c-Apochromat 1.2 NA water-immersion objective (Carl Zeiss, Jena, Germany) as previously described (Ayling et al., 2012). The confocal volume was calibrated on the day of each experiment using 20 nM Rhodamine 6G (R6G; D = 2.8 × 10^-10^ m^2^/s). The measurement volume was positioned in x and y over a flat portion of a healthy cell using a live confocal image, then approximately on the upper membrane in z. Precise z-positioning on the upper plasma membrane peak was performed using an intensity z-scan at 0.25 μm intervals for ±2 μm. Samples were excited using a 488 nm argon laser with power set to ∼0.08 kW/cm^2^ as measured at the objective. Fluorescence fluctuations were then collected through a BP505-610IR emission filter for 1 × 30 s.

Autocorrelation and PCH analysis were performed using Zen2010 Black software (Carl Zeiss, Jena, Germany). The dimensions and volume of the detection volume as well as the structure parameter (ratio of volume height to diameter) were determined from a calibration FCS read using 20 nM Rhodamine 6G, fitted to a single 3D diffusion component with a triplet state pre-exponential, as previously described (Briddon et al., 2004). Prior to AC/PCH analysis, the initial 5–10 s of data were removed where bleaching was present (indicated by a rapid decrease in the average count rate) in order to ensure that AC functions reached an asymptote at G(0) = 1. Average MOP dwell times (τ_D_) and particle number (N) were obtained from fitting of AC curves to a two-component diffusion model incorporating a pre-exponential component to account for fluorophore triple state (as per Ayling et al., 2012). This model consisted of a three-dimensional component (τ_D1_) to account for the diffusion in solution of free SNAP BG-488 label, and a two-dimensional component (τ_D2_) to account for the plasma membrane diffusion of the BG-488-labeled SNAP-MOP itself (Figure 1B). The average dwell time of free BG-488 (τ_D1_) was fixed to 32 μs in the fitting process, having been measured directly using 20nM BG-488 in HBSS. The structure parameter was also fixed to the value determined in the calibration fit. All other parameters were allowed to vary in the fitting process. Free BG-488 concentration (% τ_D1_ contribution to the AC amplitude was consistently between 10 and 15% of the total amplitude).

Average dwell time of the SNAP-MOP allowed calculation of the MOP diffusion coefficient (D_FCS_) using the equation D = ω_0_^2^/4.τ_D_, where ω_0_ was the radius of the beam waist of the detection volume determined from the calibration data and τ_D_ was the average dwell time of SNAP-MOP in the volume as determined from the AC analysis. Particle number (N) was determined as the fractional contribution of the SNAP-MOP diffusing component (τ_D2_) multiplied by the total particle number (N) as determined by the G(0) value of the fit of the AC curve. This was subsequently expressed in particles per μm^2^ (N/μm^2^), by normalizing to the area of the detection volume as projected onto a flat 2D membrane (N/πω_0_^2^).

Molecular brightness (𝜀) was determined using PCH analysis of the same fluctuation data. For PCH, the first-order correction was obtained from fitting of the R6G calibration data binned at 20 μs, and fixed to this value in subsequent fitting. Data from SNAP-MOP cells were fitted using a bin time of 100 μs. Data were fitted to either a one or two component PCH based on the goodness of fit at high photon counts per bin, visualized on a linear-log scale (Figure 1C).

### Fluorescence Recovery After Photobleaching (FRAP)

Fluorescence recovery after photobleaching measurements were performed at room temperature on a Zeiss LSM510NLO Confocor 3 inverted confocal microscope using a 40× c-Apochromat 1.2 NA water-immersion objective using the Zeiss AIM3.5 software (Carl Zeiss, Jena, Germany). After manually focusing on the basal membrane of SNAP-MOP HEK293 cells, images were scanned using 488 nm excitation, with emission collected through a BP505-610IR filter, with pinhole set at 1 Airy unit and gain and offset adjusted to fit the linear response of the PMT detectors. Images (512 × 512 pixels) were acquired continually with no averaging on zoom 2 at a rate of ∼1 frame/second. Ten pre-bleach frames were acquired before bleaching (100 ms, 100% power during 30 iterations) a circular ROI of 2.14 μm radius (area = 14.8 μm^2^) and fluorescence recovery was followed for a further 120 s.

Data were analyzed using the FRAP Wizard in Zen2010 Black software (Carl Zeiss, Germany). Fluorescence intensity within the bleached ROI of the bottom membrane was quantified over the time course of the experiment. This was background corrected using a similar sized ROI in an area of the image containing no cell and for bleaching during scanning using an ROI in a non-bleached cell. Pre-bleach frames were averaged and used as a baseline to normalize all post-bleach frames. Recovery curves were fitted to a simple exponential recovery curve to obtain a half time of recovery (t_1/2_) and a recovery plateau (Figure 1D). The mobile fraction (MF) is defined as the intensity of this plateau as a percentage of the pre-bleach control, whilst the immobile fraction (IF) is the percentage difference between intensity of the recovery plateau and the pre-bleach intensity. Diffusion coefficient (D_FRAP_) was calculated by using the equation D = ω^2^/4.τ_1/2_, where ω (μm) is the radius of the bleached area and τ_1/2_ (s) is the half-time recovery from the fitted curve in Zen2010.

### Bioluminescence Resonance Energy Transfer (BRET)

Human embryonic kidney cells were transiently transfected in a 10 cm dish. For CAMYEL, cells were transfected with 2.5 μg of SNAP-MOP and 2.5 μg of CAMYEL biosensor. For GRK2 recruitment assay, cells were transfected with 1 μg FLAG-MOP-NLuc and 4 μg GRK2-Venus. After 24 h, cells were re-plated into poly-D-lysine-coated white opaque 96-well plates (CulturPlate, PerkinElmer) and allowed to adhere overnight. BRET experiments were performed 48 h post-transfection. Cells were washed with HBSS and equilibrated for 30 min at 37°C prior to the experiment. Coelenterazine or Furimazine was added to a final concentration of 5 μM before dual fluorescence/luminescence measurement in a LUMIstar Omega plate reader (BMG LabTech). The BRET signal was calculated as the ratio of light emitted at 530 nm by Venus over the light emitted at 430 nm by Renilla luciferase 8 (RLuc8) or Nano luciferase (NLuc).

For CAMYEL assays, vehicle or DAMGO (at the indicated concentration) was added to control or PTx-pretreated cells for 10 min and baseline was measured for 4 cycles, then 10 μM forskolin was added to induce cAMP production, and the BRET signal was measured for 30 min. CAMYEL concentration response curve was constructed with the data point at 10min after cAMP stimulation by forskolin. Data were normalized to 0% inhibition (forskolin-induced cAMP production) and 100% inhibition (vehicle only without forskolin-induced cAMP production).

For GRK2 recruitment kinetic experiments, the baseline BRET ratio was measured for 4 cycles, then either vehicle (0.01% v/v DMSO) or 10 μM DAMGO was added to control or Cmpd101-pretreated cells, and the BRET signal was measured for 30 min.

### Anti-pS375 Immunocytochemistry

Human embryonic kidney cells transiently expressing FLAG-MOP were grown in a 96-well clear bottom plate (PerkinElmer). Cells were serum starved for at least 30 min and then incubated with vehicle or an EC_50_ concentration of DAMGO (1 μM) at 37°C for 5 min. Cells were then fixed in -30°C methanol for 10 min on ice. Antigen retrieval buffer was then applied, cells were heated in PBS supplemented with 10mM sodium citrate and 0.05% Tween-20 (pH 6.0) at 95°C for 20 min. Cells were then washed in PBS and incubated in blocking solution, 10% goat serum in PBS at room temperature for 30 min. Anti-FLAG mouse antibody and anti-phospho S375 rabbit antibody were diluted in blocking solution (1:1000 and 1:200, respectively) and added to cells for incubation overnight at room temperature. Cells were washed with PBS and incubated with AlexaFluor 488 (AF488) anti-mouse IgG and AlexaFluor 647 (AF647) anti-rabbit goat IgG secondary antibodies (1:1000 in blocking solution) at room temperature for 1h. Cells were washed and imaged on the Operetta High Content Imaging System capturing AF488 and AF647 channels using a 20× objective. The mean fluorescence intensity of each channel was quantified from the raw 16-bit images using the Operetta analysis software and levels of phosphorylation were expressed as a ratio of pS375 (AF647)/FLAG (AF488) immunostaining and normalized to vehicle treatment in control conditions.

### Statistical Analysis

Data representation and statistical analysis were performed using GraphPad Prism v7. FRAP and FCS data are presented as the mean ± SEM from ‘*n*’ individual cells, with the number of independent experiments also stated. For statistical analysis of clustering from PCH data, the data are represented as the mean ± SEM of the % of cells requiring a two-component fit in each independent experiment. The BRET data was quantified as indicated and represent the mean ± SEM of at least three individual experiments. Statistical significance was determined by either unpaired Student’s *t*-test or one-way ANOVA with *post hoc* Sidak’s multiple comparison analysis. *P* values for the *post hoc* tests are given in the text, whilst details of one-way ANOVA parameters are given in the relevant figure and table legends.

## Data Availability

The datasets generated for this study are available on request to the corresponding author.

## Author Contributions

AG performed and analyzed all the experiments and wrote the manuscript drafts and figures. SB, MC, and MH conceived the studies. SB supervised and designed FCS and FRAP experiments and data analysis. MH and MC supervised BRET experiments. All authors reviewed and edited the manuscript.

## Conflict of Interest Statement

The authors declare that the research was conducted in the absence of any commercial or financial relationships that could be construed as a potential conflict of interest.

## Abbreviations

AC: autocorrelation
ANOVA: analysis of variance
DAMGO: (D-Ala^2^,N-Me-Phe^4^,Gly^5^-ol)-enkephalin
FCS: fluorescence correlation spectroscopy
FRAP: fluorescence recovery after photobleaching
GPCR: G protein-coupled receptor
GRK: GPCR kinase
HEK: human embryonic kidney
MOP: μ-opioid receptor
PCH: photon counting histogram
PTx: pertussis toxin
