# Supplementary material for: GRK Mediates μ-Opioid Receptor Plasma Membrane Reorganization
Source: Front Mol Neurosci. 2019 May 1;12:104. doi: 10.3389/fnmol.2019.00104 (PMC6504784; doi:10.3389/fnmol.2019.00104)
Supplement: Supplementary file 1 [file Data_Sheet_1.pdf]

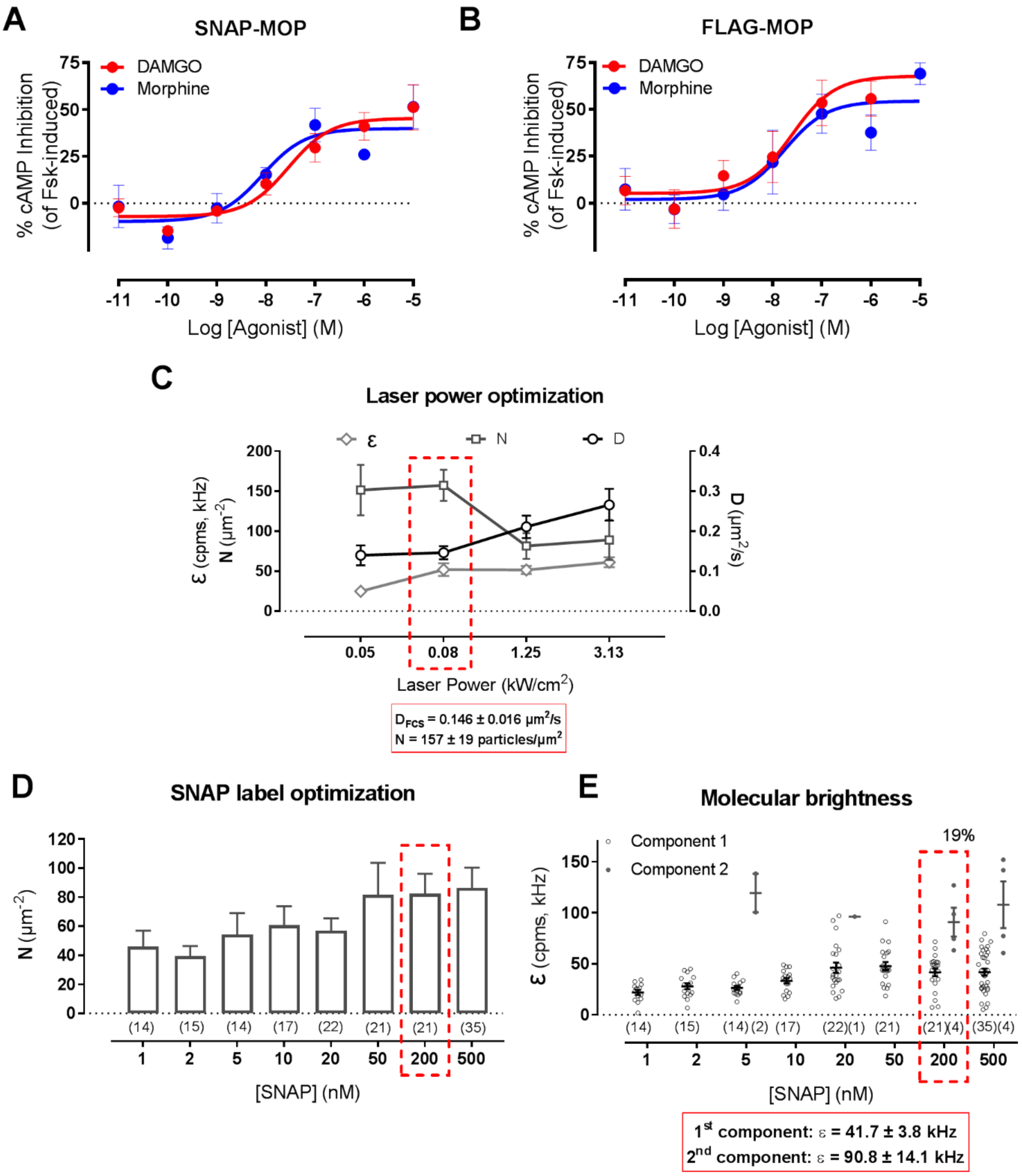

Supplementary Figure 1. Optimization and use of SNAP-MOR for FCS. Inhibition of forskolin-induced cAMP accumulation of increasing concentrations of DAMGO (red) and morphine (blue) at **(A)** the SNAP-MOP or **(B)** FLAG-MOP in a CAMYEL BRET assay (n=4). Data are shown as percentage of inhibition of cAMP induced by 10 μM forskolin (FSK) and represent mean ± SEM. **(B)** Plot of FCS parameters with increasing laser power. Molecular brightness (ε represented as diamonds) increases with increasing laser power. A decrease in particle number (N represented as squares) correlated with an increase in diffusion coefficient (D represented as circles) are indicative of photobleaching at high laser powers. The laser power chosen for subsequent experiments was 0.08 kW/cm^2 at which D and N remain consistent (n=3). Average parameters at the chosen conditions are noted. **(C)** Particle number (N) with increasing concentrations of SNAP-Surface® 488 (BG-488) dye

(n=3-6). The concentration chosen was 200 nM BG-488 since at this concentration, the particle number plateaued, indicating maximum labeling of SNAP-MOP. **(D)** Molecular brightness (ε) with increasing concentrations of SNAP-Surface® 488 (BG-488) dye (n=3-6). At high SNAP label concentrations, a second bright component was necessary for an appropriate PCH fit. Average parameters at the chosen conditions are noted.
